# *MTHFR* 677C>T Polymorphism Increases the Male Infertility Risk: A Meta-Analysis Involving 26 Studies

**DOI:** 10.1371/journal.pone.0121147

**Published:** 2015-03-20

**Authors:** Mancheng Gong, Wenjing Dong, Tingyu He, Zhirong Shi, Guiying Huang, Rui Ren, Sichong Huang, Shaopeng Qiu, Runqiang Yuan

**Affiliations:** 1 Department of Urology, The First Affiliated Hospital of Sun Yat-sen University, Guangzhou, Guangdong, 510080, China; 2 Department of Andrology, Zhongshan Affiliated Hospital of Sun Yat-sen University, Zhongshan, Guangdong, 528403, China; 3 Department of Oncology, Zhongshan Affiliated Hospital of Sun Yat-sen University, Zhongshan, Guangdong, 528403, China; 4 Department of Reproductive Center, Zhongshan Affiliated Hospital of Sun Yat-sen University, Zhongshan, Guangdong, 528403, China; 5 Department of Pharmacy, The Second People’s Hospital of Zhuhai, Zhuhai, Guangdong, 519020, China; 6 The Second General Department, Zhongshan Affiliated Hospital of Sun Yat-sen University, Zhongshan, Guangdong, 528403, China; John A. Burns School of Medicine, UNITED STATES

## Abstract

**Background and Objectives:**

Methylenetetrahydrofolate reductase (*MTHFR*) polymorphism may be a risk factor for male infertility. However, the epidemiologic studies showed inconsistent results regarding *MTHFR* polymorphism and the risk of male infertility. Therefore, we performed a meta-analysis of published case-control studies to re-examine the controversy.

**Methods:**

Electronic searches of PubMed, EMBASE, Google Scholar and China National Knowledge Infrastructure (CNKI) were conducted to select eligible literatures for this meta-analysis (updated to June 19, 2014). According to our inclusion criteria and the Newcastle-Ottawa Scale (NOS), only high quality studies that observed the association between *MTHFR* polymorphism and male infertility risk were included. Crude odds ratio (OR) with 95% confidence interval (CI) was used to assess the strength of association between the *MTHFR* polymorphism and male infertility risk.

**Results:**

Twenty-six studies involving 5,575 cases and 5,447 controls were recruited. Overall, *MTHFR* 677C>T polymorphism showed significant associations with male infertility risk in both fixed effects (CT+TT vs. CC: OR = 1.34, 95% CI: 1.23–1.46) and random effects models (CT+TT vs. CC: OR = 1.39, 95% CI: 1.19–1.62). Further, when stratified by ethnicity, sperm concentration and control sources, the similar results were observed in Asians, Caucasians, Azoo or OAT subgroup and both in population-based and hospital-based controls. Nevertheless, no significant association was only observed in oligo subgroup.

**Conclusions:**

Our results indicated that the *MTHFR* polymorphism is associated with an increased risk of male infertility. Further well-designed analytical studies are necessary to confirm our conclusions and evaluate gene-environment interactions with male infertility risk.

## Introduction

Infertility has been acknowledged as a very common health problem that affects approximately 15%-20% of couples who want to conceive [1], and almost 50% cases are because of male factors. Despite significant advancements in the male infertility diagnoses, the etiology remains unknown in almost half of all male infertile cases [2]. However, spermatogenic failure is the most common phenomenon among these cases. At present, it has been postulated that genetic abnormalities are thought to account for 15%-30% of male factor infertility, which include Y chromosome microdeletions, translocation, chromosomal aberrations and single-gene mutations [3–6]. In many infertile men, deleterious gene polymorphisms in key genes involved in testicular function, combined with environmental factors, may be responsible for the poor quality and number reduction of the sperm.

Folate is very important for the maintenance of genome integrity due to its role in DNA synthesis, repair and methylation [7, 8]. It is known that folate deficiency occur frequently, and the related hyperhomocysteinaemia is considered as a risk factor for various diseases, including infertility. Methylenetetrahydrofolate reductase (*MTHFR*) is one of the key regulatory enzymes in folate metabolism which can catalyze the reduction of 5,10-methylenetetrahydrofolate to 5-methyltetrahydrofolate, the methyl donor for homocysteine in the synthesis of methionine. Subsequently, methionine in its activated form S-adenosylmethionine is the methyl donor for DNA methylation [9]. Methylation anomalies of sperm DNA has been linked to male infertility [10]. Moreover, it has been reported that the folate metabolic pathways can be modified by polymorphisms of relevant genes such as *MTHFR* or by the action of carcinogenic elements, for example, alcohol or tobacco [11].

The *MTHFR* gene, located on the short arm of chromosome1 (1p36.3), which is composed of 11 exons [12, 13]. The change of C for T at the nucleotide position 677 of the *MTHFR* gene causes the substitution of valine for alanine in the *MTHFR* protein and a consequent reduction in enzyme activity. The *MTHFR* 677C>T variant decreases the activity of the enzyme by 35% in the presence of heterozygosis and by 70% in homozygosis [12]. Reduced enzymatic activity due to *MTHFR* polymorphisms is considered as a risk factor for many diseases, including infertility [14].

Recent years, a number of epidemiological studies have been conducted to examine the association between *MTHFR* 677C>T polymorphism and male infertility risk in diverse populations, but the results of these studies remain conflicting rather than conclusive. Some studies exhibited significantly increased risk of male infertility with *MTHFR* 677C>T, while some other studies showed nonsignificantly enhanced risk. As a result, there were five meta-analyses [15–19] performed to examine the association between *MTHFR* 677C>T polymorphism and the risk of male infertility, however, the results still inconsistent. Moreover, many new researches studied the association between male infertility risk and *MTHFR* 677C>T after the last meta-analysis researching, so an updated and high quality meta-analysis is needed.

In order to evaluate the association between the *MTHFR* 677C>T polymorphism and male infertility risk, we carried out a meta-analysis with subgroup analysis using all the eligible published data until June 19, 2014.

## Materials and Methods

### Search Strategy and Selection Criteria

According to the Meta-analysis on Genetic Association Studies Checklist (S1 Checklist), we conducted a computer-based systematic search of PubMed, EMBASE, Google Scholar and China National Knowledge Infrastructure (CNKI) without restriction on language (updated to June 19, 2014). The key words were as follows: ‘‘methylenetetrahydrofolate reductase” or “*MTHFR*”, ‘‘polymorphism” or ‘‘variant”, “infertility” or ‘‘azoospermia” or “oligoasthenoteratozoospermia” or ‘‘oligozoospermia” or “subinfertility”. In addition, we checked the references of all eligible articles which our research retrieved. For the meta-analysis, the following inclusion criteria were considered: (1) studied on human beings; (2) studies which evaluated the association between *MTHFR* 677C>T polymorphism and male infertility risk; (3) studies with case-control design; (4) sufficient published data about the size of the sample, odds ratio (OR), and their 95% confidence interval (CI). For the exclusion criteria, we provided as follows: (1) without raw data for the calculation of odds ratios (ORs) with corresponding 95% confidence intervals (95% CIs); (2) when studies with overlapping cases or controls, we included only the most recent or the largest report.

### Data Extraction

According to the inclusion and exclusion criteria, the two investigators (Tingyu He and Zhirong Shi) extracted raw data independently in order to ensure the accuracy of extracted information. For conflicting evaluations, an agreement was reached following a discussion. The following information was collected from all eligible studies showing in Table 1: the surname of the first author, date of publication, quality scores, ethnicity, sperm concentration subgroup categories, sources of controls, number of cases and controls and the *P* value of Hardy Weinberg Equilibrium (HWE). Different ethnic groups were mainly categorized as Caucasian, Asian and African. According to the sperm concentration, we divided the subgroup as azoospermia (Azoo), oligoasthenoteratozoospermia (OAT) and oligozoospermia (oligo) groups. Study designs were stratified to population-based studies and hospital-based studies.

**Table 1:** Characteristics of eligible studies in the meta-analysis of *MTHFR* 677C>T polymorphism and male infertility.

					Cases				Controls				
First author	Year	Quality scores	Group	Design	Total	CC	CT	TT	Total	CC	CT	TT	*P* HWE
**Asians**													
Park	2005	6	Total	HB	373	105	205	63	396	145	200	51	0.161
			Azoo		286	75	164	47					
			OAT		85	28	40	17					
Lee	2006	6	Total	HB	360	115	181	64	325	118	166	41	0.138
			Azoo		174	44	100	30					
			OAT		186	71	81	34					
A	2007	6	Total	NA	355	130	160	65	252	128	95	29	0.085
			Azoo		228	83	97	48					
			oligo		127	47	63	17					
Sun	2007	5	Total	PB	182	27	86	69	53	15	28	10	0.630
			OAT		22	3	11	8					
			oligo		46	10	20	16					
Yang	2010	5	Total	PB	131	34	55	42	293	98	142	53	0.901
Qiu	2011	6	Total	NA	271	75	112	84	180	63	85	32	0.720
			Azoo		158	42	66	50					
			oligo		113	33	46	34					
Safarinejad	2011	6	OAT	HB	164	58	80	26	328	144	148	36	0.825
Liu	2012	7	Total	PB	75	27	38	10	72	40	28	4	0.753
Pei	2013	7	Total	PB	290	39	138	113	90	24	47	19	0.651
Li	2014	6	Total	HB	82	14	36	32	133	36	61	36	0.340
**Caucasians**													
Ebisch	2003	7	Total	PB	77	42	28	7	113	50	48	15	0.522
Stuppia	2003	5	Total	NA	93	37	37	19	105	33	43	29	0.065
			Azoo		21	8	6	7					
			oligo		66	25	29	12					
Singh	2005	6	Total	PB	151	105	40	6	200	163	37	0	0.149
Paracchini	2006	6	Total	PB	59	11	32	16	46	18	21	7	0.830
Ravel	2009	6	Total	HB	250	118	101	31	114	49	52	13	0.887
			Azoo		70	33	31	6					
			oligo		180	85	70	25					
Murphy	2011	7	Total	PB	149	73	63	13	182	94	73	15	0.876
Gupta	2011	5	Total	HB	522	378	116	28	315	251	58	6	0.228
			Azoo		68	49	15	4					
			OAT		65	41	23	1					
Chellat	2012	5	Total	NA	74	31	33	10	84	36	38	10	0.995
			Azoo		46	20	19	7					
			OAT		28	11	14	3					
Weiner	2013	6	Total	PB	271	129	116	26	301	153	115	33	0.112
			Azoo		98	49	41	8					
			oligo		82	40	31	11					
Naqvi	2014	5	Total	NA	637	447	154	36	364	275	79	10	0.145
			Azoo		49	34	11	4					
			OAT		65	39	24	2					
			oligo		37	23	12	2					
Mfady	2014	6	Total	HB	150	67	63	20	150	74	67	9	0.221
**Africans**													
Eloualid	2012	5	Total	NA	257	152	88	17	690	351	286	53	0.617
			Azoo		110	65	37	8					
			oligo		147	87	51	9					
**Not mentioned**													
Gava (37)	2011	7	Total	PB	156	81	60	15	233	167	53	13	0.003
			Azoo		49	27	15	7					
			oligo		107	54	45	8					
Gava (49)	2011	7	Total	PB	133	66	51	16	173	136	27	10	0.000
			Azoo		55	35	14	6					
			oligo		78	31	37	10					
Vani	2012	6	Total	HB	206	158	42	6	230	188	42	0	0.128
Camprubí	2013	6	Total	PB	107	47	43	17	25	8	15	2	0.172
			oligo		23	12	9	2					

Azoo-azoospermia; OAT-oligoasthenoteratozoospermia; oligo-oligozoospermia

HB, hospital-based controls; PB, population-based controls

HWE, Hardy Weinberg Equilibrium

NA, not available

### Quality assessment

Three authors (Guiying Huang, Rui Ren and Sichong Huang) assessed the study quality independently based on the Newcastle-Ottawa Scale [20], which uses a star rating system to judge the methodological quality. A full score is 9 stars, and a score range 5 to 9 stars is considered to be a generally high methodological quality while a score range 0 to 4 is considered to be a poor quality [21]. The quality of all included studies was summarized in Table 2. Any disagreements on the NOS score of the studies were resolved through a comprehensive reassessment by the other authors and only high quality studies can be included in our meta-analysis.

**Table 2:** Quality assessment based on the Newcastle-Ottawa Scale of studies included in this meta-analysis^a^.

First author	Year	Adequate definition of case	Representativeness of cases	Selection of control	Definition of control	Control for important factor or additional factor^b^	Exposure assessment	Same method of ascertainment for cases and controls	Nonresponse rate	Total quality scores
Ebisch	2003	★	★	★	★	★★		★		7
Stuppia	2003	★	★			★★		★		5
Park	2005	★	★		★	★★		★		6
Singh	2005	★	★	★		★★		★		6
Lee	2006	★	★		★	★★		★		6
Paracchini	2006	★	★	★	★		★	★		6
A	2007	★	★		★	★★		★		6
Sun	2007	★	★	★	★			★		5
Ravel	2009	★	★		★	★★		★		6
Yang	2010	★	★	★	★			★		5
Gava (37)	2011	★	★	★	★	★★		★		7
Gava (49)	2011	★	★	★	★	★★		★		7
Safarinejad	2011	★	★		★	★★		★		6
Murphy	2011	★	★	★	★	★★		★		7
Qiu	2011	★	★		★	★★		★		6
Gupta	2011	★	★		★	★		★		5
Eloualid	2012	★			★	★★		★		5
Chellat	2012	★	★		★	★		★		5
Vani	2012	★	★		★	★★		★		6
Liu	2012	★	★	★	★	★★		★		7
Camprubí	2013	★	★	★	★	★		★		6
Pei	2013	★	★	★	★	★★		★		7
Weiner	2013	★	★	★		★★		★		6
Naqvi	2014	★	★		★	★		★		5
Mfady	2014	★	★		★	★★		★		6
Li	2014	★	★		★	★★		★		6

^a^A study can be awarded a maximum of one star for each numbered item except for the item Control for important factor or additional factor.

^b^A maximum of two stars can be awarded for Control for important factor or additional factor.

### Statistical Analysis

The association between *MTHFR* 677C>T polymorphism and the male infertility risk were estimated by pooled ORs with 95% CI. The statistical significance of the pooled ORs was examined by *Z* test and *P* (two-tailed) <0.05 was considered statistically significant. A chi-square test was used to examine the deviation from HWE for controls and *P* value<0.05 signified a departure from HWE. In this meta-analysis, heterogeneity between studies was assessed by the *I* ^*2*^ test and which was considered statistically significant with *I* ^*2*^>50% [22]. A fixed (the Mantel-Haenszel method) [23] or random effects model (the DerSimonian and Laird method) [24] was used to calculate pooled effect estimates in the absence (*I* ^*2*^≤50%) or presence (*I* ^*2*^>50%) of heterogeneity, respectively. When heterogeneity between studies is absent, these two models provide similar results, if not, the random effects model is more appropriate. Subgroup analysis was performed by ethnicity, sperm concentration and the control sources. In addition, to assess the stability of results, sensitivity analysis was performed by omitting one study at a time and calculating the overall homogeneity and effect size. As publication bias was always concerned in meta-analysis, an evaluation of which was carried out with funnel plot and Egger’s test (*P*<0.05 was significant publication bias) [25]. The statistical analysis was performed with STATA statistical software (Version 12.0; Stata Corporation, College Station, TX, USA).

## Results

### Study characteristics

**Fig 1:**
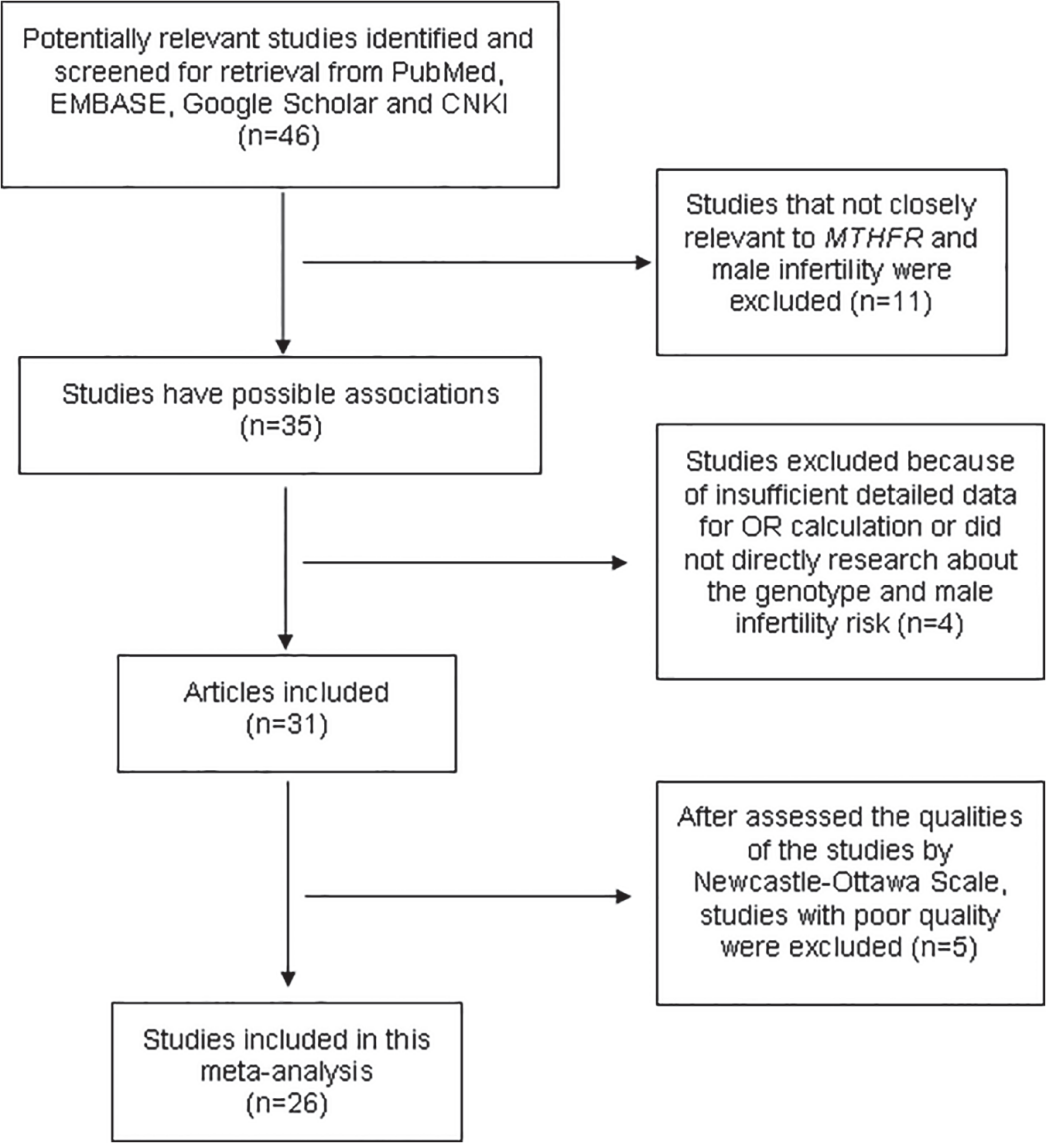
Flow diagram of study selection.

Through a literature searching, initially a total of 46 potentially relevant publications were indentified. Out of these, eleven studies were eliminated because they did not investigate the association between the *MTHFR* 677C>T polymorphism and male infertility risk. After data extraction, we excluded 4 articles from the meta-analysis. One of them had no controls [26] while one of them had no cases [27], besides, the other two studies were excluded as one did not provide detailed information needed for OR calculation [28] while the last one did not directly research about the genotype and male infertility risk [29]. Hence, we obtained 31 relevant articles that examined the association between *MTHFR* 677C>T and male infertility risk. Among of them, five studies were excluded because of the poor quality, which was evaluated by Newcastle-Ottawa Scale [30–34]. Therefore, only 26 studies qualifying our strict selection criteria were involved in the meta-analysis [11, 16, 19, 35–57] (Fig. 1). We established a database of the extracted information from each eligible article (Table 1). The total data for this analysis included 5,575 cases and 5,447 controls for *MTHFR* 677C>T polymorphism. All the researches contained in this meta-analysis are case-control studies. Of the 26 studies included in the meta-analysis, there were 10 studies of Asians, 11 studies of Caucasians, 1 study of African and 4 studies did not mention. According to the control sources, general populations were used as controls in 12 studies whereas hospital patients were used in 8 studies and 6 studies did not mention. The genotype distributions among the controls of all studies followed HWE except for two studies [37, 49].

### Overall analysis

**Fig 2:**
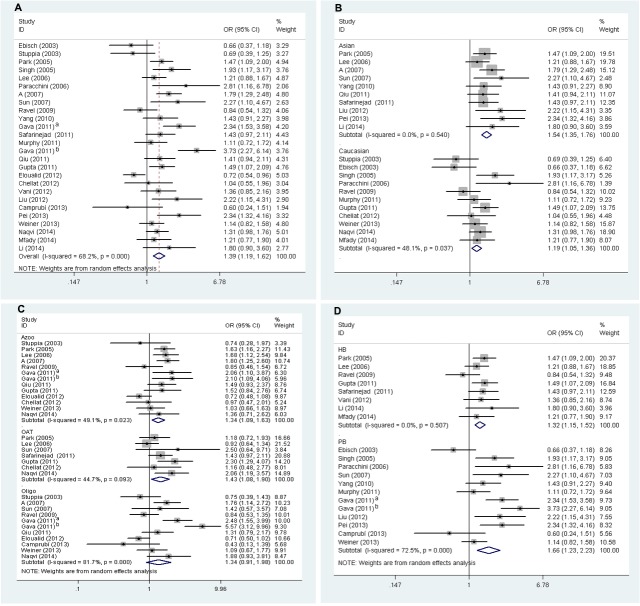
Forest plots of *MTHFR* 677C>T polymorphism and male infertility risk (CT+TT vs. CC). [**A** for overall populations; **B** for ethnicity subgroup; **C** for sperm concentration subgroup; **D** for control sources subgroup]. The squares and horizontal lines correspond to the study-specific OR and 95% CI. The area of the squares reflects the study-specific weight (inverse of the variance). Diamonds represent the pooled OR and 95% CI. ^a^the 37^th^ reference, ^b^the 49^th^ reference

*MTHFR* 677C>T polymorphism showed significant associations with male infertility risk in both fixed effects (CT+TT vs. CC: OR = 1.34, 95% CI: 1.23–1.46, *P* = 0.000, *I* ^*2*^ = 68.2%; TT vs. CT+CC: OR = 1.60, 95% CI: 1.41–1.81, *P* = 0.000, *I* ^*2*^ = 36.9%; TT vs. CC: OR = 1.76, 95% CI: 1.53–2.01, *P* = 0.000, *I* ^*2*^ = 54.0%; T vs. C: OR = 1.32, 95% CI: 1.24–1.41, *P* = 0.000, *I* ^*2*^ = 71.5%) and random effects models (CT+TT vs. CC: OR = 1.39, 95% CI: 1.19–1.62, *P* = 0.000, *I* ^*2*^ = 68.2%, Fig. 2A; TT vs. CT+CC: OR = 1.58, 95% CI: 1.34–1.88, *P* = 0.000, *I* ^*2*^ = 36.9%; TT vs. CC: OR = 1.80, 95% CI: 1.44–2.24, *P* = 0.000, *I* ^*2*^ = 54.0%; T vs. C: OR = 1.36, 95% CI: 1.20–1.53, *P* = 0.000, *I* ^*2*^ = 71.5%).

### Ethnic origin

When stratified by ethnicity, the same associations between the *MTHFR* 677C>T polymorphism and male infertility were found in Asians (CT+TT vs. CC: fixed effects model, OR = 1.54, 95% CI: 1.35–1.76, *P* = 0.000, *I* ^*2*^ = 0.0%) and Caucasians (CT+TT vs. CC: fixed effects model, OR = 1.19, 95% CI: 1.05–1.36, *P* = 0.007, *I* ^*2*^ = 48.1%, Fig. 2B).

### Sperm concentration subgroup

Subgroup analyses were also performed for azoospermia (Azoo), oligoasthenoteratozoospermia (OAT) and oligozoospermia (oligo) groups (Fig. 2C). Studies without the genotype frequencies for these subgroups were excluded. Finally, the total number of cases and controls for Azoo, OAT and oligo were 1,412 and 3,532, 615 and 1,865, 1,006 and 2,490, respectively. The results showed enhanced risks of male infertility with the *MTHFR* 677C>T were acquired both in Azoo (CT+TT vs. CC: fixed effects model, OR = 1.36, 95% CI: 1.18–1.55, *P* = 0.000, *I* ^*2*^ = 49.1%) and OAT (CT+TT vs. CC: fixed effects model, OR = 1.35, 95% CI: 1.11–1.64, *P* = 0.003, *I* ^*2*^ = 44.7%) subgroups. Whereas, no significant association was observed in oligo subgroup (CT+TT vs. CC: random effects model, OR = 1.34, 95% CI: 0.91–1.98, *P* = 0.138, *I* ^*2*^ = 81.7%).

### Control sources

When considered the sources of the control groups, five studies were excluded for unclear source of controls. Dramatically elevated risks were found both in population-based (CT+TT vs. CC: random effects model, OR = 1.66, 95% CI: 1.23–2.23, *P* = 0.001, *I* ^*2*^ = 72.5%) and hospital-based controls (CT+TT vs. CC: fixed effects model, OR = 1.32, 95% CI: 1.15–1.52, *P* = 0.000, *I* ^*2*^ = 0.0%) (Fig. 2D).

### Sensitivity analyses

To assess the effect of individual study on all subjects and subgroups, the sensitivity analyses were performed by excluding one study each time. If the exclusion of any single study did not alter the significance of the final decision, it suggested that the outcomes were robust. However, our corresponding pooled ORs were not materially altered in all subjects and all subgroups. In addition, when excluding the two studies [37, 49] which did not follow HWE, the pooled ORs for all subjects still did not change remarkably (data not shown). Therefore, the results of sensitivity analyses confirmed the stability for our results in this meta-analysis.

### Publication bias

**Fig 3:**
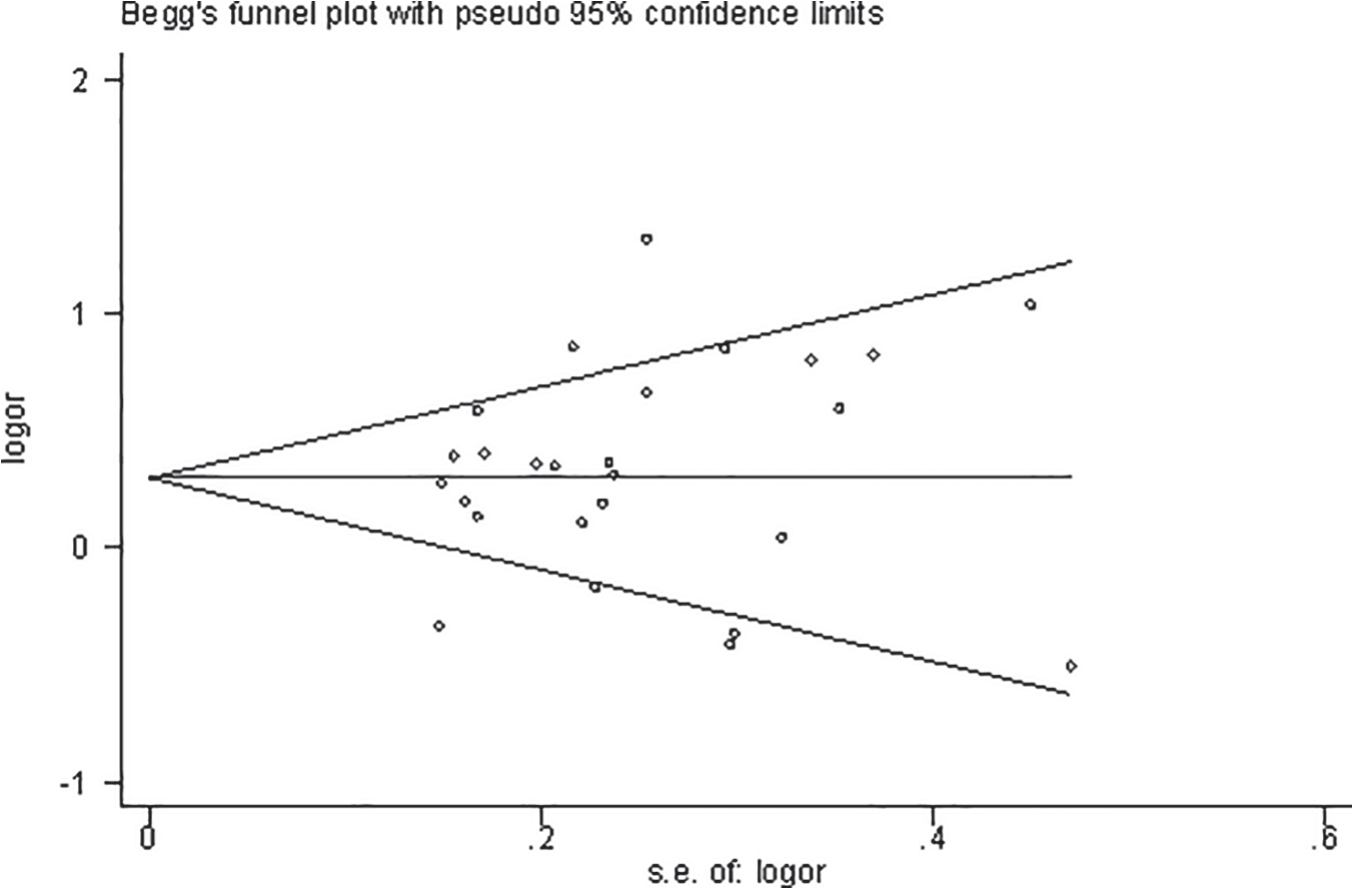
Funnel plot for publication bias of *MTHFR* 677C>T and infertility risk (CT+TT vs. CC) in overall populations. The horizontal line in the funnel plot indicates the random-effects summary estimate. Sloping lines indicate the expected 95% CI for a given SE.

For the diagnosis of publication bias, both Begg’s Funnel plot and Egger’s test were performed in this meta-analysis. The shape of the funnel plot was symmetrical and the *P* value of Egger’s test also suggested that there was no evidence of publication bias (*P* = 0.339; Fig. 3).

## Discussion

Male infertility is a heterogeneous disease, with various genetic and environmental factors contributing to the impairment of spermatogenesis. Spermatogenesis is a complex process that governed by a tightly controlled series of gene expression events [58] and is associated with folate metabolism [59–61]. A possible candidate gene for genetic susceptibility to spermatogenic failure is *MTHFR*. *MTHFR* is an important regulatory enzyme in folate metabolism, DNA synthesis and remethylation reactions [62, 63]. It is suggested that the *MTHFR* might play an important role in spermatogenesis because of its higher activity in testes than in other major organs in the adult mouse [64]. In 2001, Bezold et al. [31] first reported the association between *MTHFR* 677C>T mutation and the male infertility risk. Subsequently, many epidemiological studies have been addressed to investigate the association between *MTHFR* 677C>T polymorphism and the risk of male infertility during the past decades, but the findings were inconsistent. Consequently, five meta-analyses [15–19] were conducted to exam the association between male infertility risk and the *MTHFR* 677C>T polymorphism, however, the results of them were controversial. In addition, the data of last meta-analysis [19] was updated two years ago (Apr. 2012). During these two years, many studies investigated the association between the *MTHFR* 677C>T polymorphism and male infertility risk were published. Therefore, in order to derive a powerful estimate of the male infertility risk associated with the *MTHFR* 677C>T polymorphism, we carried out the present meta-analysis.

The present meta-analysis, including 5,575 cases and 5,447 controls from 26 published studies, explored the association between the *MTHFR* 677C>T polymorphism and male infertility risk. The numbers of contained studies, the cases and controls in this meta-analysis are much more than the prior five meta-analyses. In addition, we did the quality assessment for the studies by Newcastle-Ottawa Scale while all the former meta-analyses did not do. Only the study had good quality of which score was more than 5 stars, then it could be included in the meta-analysis. Hence, our study made a more detailed and convincible evaluation than all the prior meta-analyses did. Overall, we find the *MTHFR* 677C>T variant genotype were significantly associated with male infertility risk based on both fixed effects and random effects models, and which was consistent with the results of Tüttelmann et al. [15], Gupta et al. [16] and Wu et al. [17], but inconsistent with the results of Wei et al. [18] and Weiner et al. [19]. All the characteristics and results of the present study compared with the former five meta-analyses were summarized in Table 3.

**Table 3:** Characteristics and results of the present study compared with the former five meta-analyses.

First author	Year of publication	Time of data updated	No. of studies	No. of cases	No. of controls	Quality assessment	Overall results	Asians	Caucasians	Azoo	OAT	Oligo	PB	HB
Tüttelmann F	2007	NA	8	1843	1791	No	+	NA	NA	NA	NA	NA	NA	NA
Gupta N	2011	Mar. 2011	14	3094	2877	No	+	NA	NA	+	−	+	NA	NA
Wu W	2012	Sep. 2010	9	2275	1958	No	+	+	−	+	−	NA	NA	NA
Wei B	2012	Jan. 2011	11	2217	2312	No	−	+	−	NA	NA	NA	NA	NA
Weiner AS	2014	Apr. 2012	13	2972	3436	No	−	NA	NA	−	NA	−	NA	NA
The present study	NA	Jun. 2014	26	5575	5447	Yes	+	+	+	+	+	−	+	+

Azoo-azoospermia; OAT-oligoasthenoteratozoospermia; Oligo-oligozoospermia

HB, hospital-based controls; PB, population-based controls

+, positive result

−, negative result

NA, not available

As heterogeneity for *MTHFR* 677C>T polymorphism among all the studies was significant, we conducted the subgroup analyses. When stratified by ethnicity, our results indicated that significant male infertility risks of people with *MTHFR* 677C>T polymorphism both in Asians and Caucasians. We are the first one that reported significant association between *MTHFR* 677C>T polymorphism and male infertility in Caucasians. Moreover, the heterogeneity was effectively decreased or removed after subgroup analyses by ethnicity. Therefore, as the differences of genetic backgrounds among different populations, one main reason for heterogeneity in this meta-analysis may be the ethnicity.

According to the sperm concentration, we found remarkable associations between Azoo or OAT subgroup and *MTHFR* 677C>T polymorphism in the present study. While, no strong association was observed in oligo subgroup. Compared with the former meta-analyses, we are the first one reported the *MTHFR* 677C>T polymorphism has a dramatically increased risk in male infertility susceptibility in OAT subgroup and the sensitivity analysis showed the stabilization of this result. However, as the extreme heterogeneity existed in oligo subgroup, the result of it may be less powerful and we should treat it carefully. Further studies based on oligo and *MTHFR* 677C>T are necessary to confirm the association.

In addition, when stratified by the sources of the control groups, our results showed that the *MTHFR* 677C>T polymorphism were dramatic associated with male infertility risk both in population-based and hospital-based controls. At present, it is reported that the *MTHFR* 677C>T polymorphism could influence the susceptibility to some common diseases, such as type 2 diabetes [65], coronary heart disease [66] and colorectal cancer, et al. [67]. Thus, the data on hospital-based controls may have a high risk of inducing unreliable result because it may not always represent the general population indeed, especially when the genotype under investigation are expected to affect other diseases. However, we obtained consistent results between population-based and hospital-based controls.

In recent years, the application of the genome-wide association study (GWAS) in many types of diseases has exploded. Until now, there are twelve GWASs about male infertility, and two of them [68, 69] mentioned the *MTHFR* 677C>T polymorphism. However, concerned with the male infertility and *MTHFR* 677C>T polymorphism, the results of these two GWASs were inconsistent with our meta-analysis. The reasons of this phenomenon are possibly as follows: (1) These two GWASs both conducted by Aston KI only included Caucasians of European decent, while we included men of other ethnicities. SNP frequencies vary widely between ethnic groups, for example, the frequency of the *MTHFR* 677C>T allele varies significantly among populations, ranging from 30% to 40% in Europe and America to 5% to 10% in Africa and Sri Lanka [70, 71]; (2) The small sample size both of these two GWASs (Aston et al., 2009: 80 controls, 92 cases; Aston et al., 2010: 158 controls, 282 cases) limited the interpretation of the results. However, a large portion of the heritability of complex diseases has not been well explained by GWASs [72]. About missing heritability, one plausible explanation may be the loss of low-frequency variants, which are not well captured by current genotyping arrays [73]. Another explanation is that the common variants of small effect sizes, do not reach the thresholds of genome wide significance in GWASs [74].

Although several previous meta-analyses have researched the association between male infertility risk and *MTHFR* 677C>T polymorphism, our study was more rigorous and comprehensive. First, more up-to-date studies (26 studies) that contained a substantial number of cases and controls were pooled from published studies, which dramatically increased the statistical power of the analysis. Second, the quality of studies included in this meta-analysis was satisfactory, as we first used the Newcastle-Ottawa Scale to assess the quality of the articles. When the study met our strict criteria, it could be enrolled in. Third, we are the first that showed the significantly increased risk between *MTHFR* 677C>T polymorphism and male infertility in Caucasians and OAT subgroup. Also, for the first time we performed in detail to check into the association between male infertility risk and *MTHFR* 677C>T polymorphism according to the control sources. Our results showed obvious higher male infertility risk in both population-based and hospital-based controls.

Nonetheless, there are some limitations should be addressed in this meta-analysis. First of all, the heterogeneities for *MTHFR* 677C>T polymorphism among all the studies was dramatic. Even though we conducted subgroup analyses and the heterogeneity was efficiently decreased or removed in some subgroups, it still existed in the population-based controls and oligo subgroup. Several reasons could explain the significant heterogeneity. First, lifestyle factors especially intake of folate and B-vitamins may play a role. For instance, deficient intake of folate and vitamin B12 is prevalent in India [75–77]; in contrast, sufficient intake of folate is prevalent in France [78], Korea [11] and Italy [79]. Second, due to the various genotyping methods used in the studies, the genotyping error is hard to avoid. However, when we eliminated the studies which did not follow the HWE, our results did not change statistically. Third, it has been shown that there were other underlying confounding factors in the included studies, such as gene-environment interaction, or selection bias, or chance [80, 81]. Although evidence of heterogeneity exists, our overall results used by fixed effects models were consistent with which used by random effects models. Moreover, the sensitivity analyses results showed that all of our results were stable and certain. Secondly, as the use of unadjusted data, some potential confounding factors, such as age, sex and residence might slightly modify the effective estimates. Hence, a more precise evaluation according to the adjusted data is needed. Finally, as only published studies were retrieved in the present meta-analysis, the selection bias may have occurred, even though the funnel plot and Egger’s test have not shown publication bias. Also, the number of published studies was not large enough for a comprehensive analysis, particularly for some subgroups.

## Conclusions

In summary, our meta-analysis suggested that the *MTHFR* 677C>T polymorphism is associated with an enhanced risk of male infertility, supporting the hypothesis that the *MTHFR* 677C>T may be a potential cause of male infertility. Specifically, increased infertility risks with *MTHFR* 677C>T were observed in overall analysis and most subgroups, except oligo subgroup. However, large scale, well-designed and high quality epidemiological studies will be required to confirm our findings in the future.

## Supporting Information

S1 Checklist (DOC) (DOCX)
